# Rumination and Worry Selectively Modulate Total Calorie Consumption within an Online, Nudge Tactic Paradigm

**DOI:** 10.3390/bs12030067

**Published:** 2022-03-02

**Authors:** Timothy M. Eschle, Sarah P. Wale, Dane McCarrick

**Affiliations:** 1 Department of Psychology, School of Education and Social Sciences, University of the West of Scotland, Paisley PA1 2BE, UK; sarahpwale@gmail.com; 2 School of Psychology, Faculty of Medicine and Health, University of Leeds, Leeds LS2 9JT, UK; psdjm@leeds.ac.uk

**Keywords:** rumination, worry, perseverative cognition, snack choice, health behaviours

## Abstract

Rumination and worry, collectively referred to as perseverative cognition, have been implicated in the increased engagement of several health risk behaviours. The current study aimed to investigate the potential influencing role of these repetitive negative thought cognitions in an online snack paradigm. Participants were randomly assigned to either an even condition (a 3:3 ratio of ≤101 kcal and >201 kcal snacks) or an uneven condition (a 4:2 ratio in favour of ≤101 kcal snacks). Upon the presentation of six images of sweet treats, participants were asked to choose the snack they most wanted to consume “right now”, before completing the Ultra-Brief Penn State Worry Questionnaire (UB-PSWQ) and the brief (5-item) Ruminative Response Scale (RRS). The results showed that the reduced availability of higher calorie snacks significantly improved both snack choice and total calorie consumption. However, despite rumination and worry having no influence on the snack type chosen, higher levels of rumination still led to significantly higher overall calorie consumption. Although, contrary to predictions, higher levels of worry conversely led to significantly lower overall calorie consumption. This study adds to the growing work in the role of perseverative cognition and food consumption, which may aid in informing public health strategies. Further exploration is needed to assess whether rumination directly induces unhealthy eating behaviours or simply exacerbates them.

## 1. Introduction

The World Health Organization (WHO) predicts that at least 2.8 million deaths globally are a result of being overweight and obese [1], while the UK government estimates that being overweight and obese-related ill health costs the national health service ~£6.1 billion annually [2]. Excess calorie consumption is the primary contributor to obesity and its comorbidities [3,4], and thus, interventions employed by the UK government, and associated shareholders, have been targeted towards calorie reduction [2]. One of the more expeditious environmental interventions aimed at reducing calorie consumption and improving food selection is that of choice architecture. Indeed, varying the availability of unhealthy food choices in particular has shown effective in significantly improving food choice in a series of online, laboratory and field-based investigations [5,6,7,8,9,10]. Axiomatically, there are a range of individual differences (e.g., demographic, trait and affective factors) that have been shown to influence food choice, both within and independently of these behavioural paradigms [9,10,11,12,13]. Although, among the multifaceted causes of surplus calorie consumption, emotional eating (particularly in response to negative emotions and stress) has been shown to be a principal catalyst [14,15].

Stress has been shown to modify food consumption in several ways, including increases in hedonic hunger, snack consumption and a higher preference for calorie dense foods [16,17,18,19,20], while also leading to an inverse effect on healthy eating behaviours such as reduced fruit, vegetable intake and main meal consumption [20,21].

This could be a result of several factors, namely the modulation of appetite related hormones and or of the hypothalamic-pituitary-adrenal (HPA) axis functioning (see [22] for a review). Regarding the latter, the perseverative cognition (PC) hypothesis postulates that repetitive negative thinking, in the form of rumination and worry, leads to prolonged hypothalamic activation and, consequently, the exacerbation of both poorer stress-induced health outcomes [23,24]. This is supported by observations that both worry and rumination are associated with physiological reactions related to the stress response [25,26]. Moreover, the emerging literature available has revealed that specific components and subcomponents of PC are associated with elevated engagement in health risk behaviours [27,28]. For instance, a recent meta-analysis has found that both rumination and worry were significantly related to sleep duration and quality [29], while brooding (the maladaptive component of rumination) has been found to be instigated in the worsening of smoking, alcohol consumption and unhealthy eating behaviours [27,28].

However, further research is still needed to elucidate whether PC and/or its subcomponents can modulate health risk behaviours in their own right [28]. Equally, given that many of these health risk behaviours are mediated by impulse and momentary choice, consideration should be assigned to the potential of the use of acute behavioural interventions to potentially mitigate the harmful effects. Of interest here is the use of choice architecture in the form of relative reductions in unhealthier options to guide improved food choice selection when the total number of items remains constant. Naturally, such investigations should focus specifically on relevant food options that are most likely to be consumed by stressed individuals (e.g., comfort food). Similarly, the alternative (i.e., healthier) options should also be of the same variety of products but with a noticeable health-related improvement to their nutritional content. This would allow for appropriate measurement of how choice architecture could improve an already poor food selection in individuals that are most likely to make adverse food decisions.

In this vein, in a previous pilot investigation conducted by the current research team, we aimed to establish whether state and or trait PC could influence snack choice within a forced choice, nudge paradigm [30]. Participants were randomly allocated to an uneven or an even condition before completing a snack choice task. Those in the uneven condition were randomly presented with a higher number of front of packet lower calorie snacks (≤99 kcal) in contrast to the even condition (4:2 and 3:3 respectively), which displayed an even presentation of lower and high (>200 kcal) snacks. Participants were instructed to choose the snack that they most wanted to consume “right now”, before completing a series of scales regarding their trait and momentary negative thought patterns. The results showed that those in the uneven condition were more likely to opt for lower calorie snacks choices, relative to the even group. However, despite a trend towards an association between state rumination and higher calorie snacks, both state and trait PC measures did not significantly influence snack choice.

There was one notable limitation to this work. The focus was on the quality of the food chosen (in this case, its calorie content) rather than quantity (i.e., how many items of the chosen snack the participant wanted to consume). This is particularly important given that stress leads not only to increased preference for calorie dense foods but also elevated amounts of consumption of such foods. In addition, it is axiomatic that calorie consumption increases, regardless of food type, when individuals are allowed to eat ad libitum as opposed to restricted eating paradigms. Consequently, the task at hand may have inadvertently limited the measuring of any potential effects of PC on total food consumption. Going forward, the paradigm needs to be updated to employ an additional measure that includes gauging quantity of snacks and subsequent predicted overall calorie intake. The current study aimed to build upon our previous pilot investigation with the aforementioned changes to the snack choice paradigm in order to assess whether rumination and worry can module both snack choice and quantity of snacks chosen. The present study also aimed to assess the capacity of increased availability of lower calorie snacks to improve snack choice and the amount of snacks chosen.

It was predicted that higher levels of rumination and worry would increase the likelihood of choosing a higher calorie snack (Hypothesis 1a) and increase the total number of hypothetical calories consumed (Hypothesis 2a). It is also predicted that the uneven condition (as characterized by a higher presentation of lower calorie snacks in a 4:2 ratio) will increase the likelihood of opting for a lower calorie snack option (Hypothesis 1b) and lower total hypothetical calorie consumption (Hypothesis 2b) relative to the even group (3:3 ratio of presentation of low and higher calorie snacks).

## 2. Materials and Methods

### 2.1. Design

The current online study was conducted through Qualtrics. The Qualtrics randomizer tool was employed to randomise the participants into one of two conditions in a double-blind design. Participants were allocated to either the experimental condition (uneven condition) where participants were presented with a ratio of 4:2 in preference of low-calorie snacks (≤101 kcals), or a control condition (even condition) with an even presentation of 3:3 for both low and high calorie snacks (>201 kcal). The key predictors were rumination, as measured by the Ruminative Response Scale (RRS), and worry, as determined by the score on the Ultra-Brief Penn State Worry Questionnaire (UB-PSWQ). These scales are described in further detail below.

### 2.2. Participants

A total of 338 participants were recruited online through social media posts and relevant survey share forums. The inclusion criterion for participation was that individuals must be aged 18 years or over and to be free of a diagnosis of a mental health and or eating disorder. Full details of the demographics of the full sample and individual conditions are available in Table 1 below. To assess for any potential differences in demographic variables across the two groups, a series of student *t*-tests was conducted. From this, no significant differences were identified across the two groups for any of the demographic variables (all *p* > 0.10).

### 2.3. Materials

#### 2.3.1. Snack Choice Task

As with the previous pilot investigation [30], this task presented participants with six images of sweet snack items simultaneously and in a randomised order on screen. Those in the even condition were faced with an array of four low calorie snack items and two higher calorie items, while those in the even condition were presented with three low calorie and three higher calorie snacks. All items were displayed with the name of the relevant snack, alongside the weight (in grams) and calorie content of the snack. The instructions provided to the participants was to select the snack item that they would most like to consume “right now”. Upon their selection, participants were presented with their chosen snack on screen and then asked to quantify how many of this item (and relevant serving) that they would like to consume. Responses were measured on a 1–8 scale, with a further “other” option to allow participants to manually report a number of their choosing (above 8 if applicable). An illustrative example of this task can be found in Supplementary Materials S1. Although this may be deemed an arbitrary measure, it is important to note that higher scores still infer the intensity of desire to consume that volume of food, and they also may be an accurate representation of how many may be consumed in more ecologically valid conditions. Such a technique has been employed successfully in other studies [31].

#### 2.3.2. Stimuli

All stimuli and their accompanying information (weight, serving and calorie content) was retrieved (with permission) from the “food.pics” database [32,33]. The final stimuli chosen for the uneven (4:2) condition were a chocolate cookie (97 kcal; 19 g), marzipan chocolate bite (91 kcal; 17 g), marshmallows (101 kcal; 30 g), liquorice wheels (75 kcal; 20 g), donut (227 kcal; 50 g), jelly sweets (274 kcal; 80 g). The same stimuli were employed for the even condition, with the exception that the licorice wheels were exchanged in favor of the presentation of a cupcake (275 kcal; 80 g). All stimuli were reported to have good palatability levels. In line with the usage statement of the license agreement, the catalog numbers of each of the stimuli used in this study has been provided in Supplementary Materials S1.

#### 2.3.3. Questionnaires

##### The 3-Item Ultra-Brief Penn State Worry Questionnaire (UB-PSWQ-3)

The original 16-item Penn State Worry Questionnaire (PSWQ) is the leading and most commonly utilized self-report measure of trait worry [34]. Here, participants are required to respond to statements with regards to how they typically think on a 1 (“not at all typical of me”) to 5 (“very typical of me”) scale. In the current investigation, the 3-item UB-PSWQ version was employed. This version has shown comparable internal consistency, convergent validity and discriminant validity with the original 16-item scale [35].

##### The Brief (5-Item) Ruminative Response Scale (RRS)

The 22-item RRS [36] was first revised into 10 items by Treynor and colleagues [37] reflecting the two subscales of rumination, i.e., brooding and reflection (5 items each). The present study utilized the brief (5-item) RRS which has shown acceptable internal consistency and has been shown to correlate well with both the original 22-item RSS (r = 0.88) and also the brooding subscale of the 10-item RRS (r = 0.70) [38]. Participants responded to each item on a 1 (“almost never”) to 4 (“almost always”) scale, detailing how they generally behave. As with the use of the UB-PSWQ-3, this shortened scale allows for time-efficient responses from participants, without compromising psychometric soundness [38].

##### Visual Analogue Scale (VAS)

The employment of a VAS can efficiently and effectually gauge a wide range of human states. Three separate VASs were used to measure self-reported momentary hunger and the relative intensity of both worry and ruminating thought patterns (respectively) that the participants felt they had experienced that day. For all VASs, participants were presented with the relevant statement (e.g., “How hungry are you?”) before being requested to indicate their response via a 100 mm line on the screen; describing the two extremes of the specific mood being measured, anchored from left (0) to right (100). For hunger, participants were asked to consider how they were feeling at this very moment in time, while for the rumination and worry assessments, participants responded to how much they had had these negative thought cognitions “today”. For perspective, the instructions for all VASs was that the two ends of the scale represent the two extremes. For instance, 0 denoted “the least” the participant had ever felt such a sensation and 100 indicated the most they had ever experienced this in their entire life. The wording for the rumination and the worry VASs were adapted from [39,40]. An example of these three VAS can be found in Supplementary Materials S2.

### 2.4. Procedure

Upon clicking on the link to the survey, participants were first briefed on the nature and inclusion criteria of the study via an on-screen information sheet. Eligible and willing participants then provided informed consent before beginning the survey. After, participants then completed a series of demographic questions before completing the snack choice task. Following this, participants responded to the hunger VAS before completing the brief RRS and the 3-item UB-PSWQ and the two remaining VASs. The order of the survey was so, to avoid any undue influence of the statements within the scales or prompting the aims of the study into the responses made in the food choice paradigm. Once the survey was complete, the participants were presented with a debrief form. 

This protocol was approved by the School of Education and Social Sciences at the University of the West of Scotland (Approval Number: 16591; 13877).

### 2.5. Analysis

Data were analysed in SPSS (version 25). A logistical regression was first conducted to address Hypothesis 1a and 1b respectively; assessing the role of the worry, rumination and condition on snack choice. The relevant outcome variable for this analysis was membership to either choosing a low calorie (≤101 kcal) or a high calorie (>201 kcal) option. Results of this analysis are reported in odds ratio. To address Hypothesis 2a and 2b, a multiple regression was conducted. The outcome variable of interest here was the total number of calories calculated via the calories of the item chosen multiplied by the quantity selected. Self-reported hunger was included in both analyses as a covariate. All relevant assumptions for the two regressions were satisfied.

## 3. Results

### 3.1. Treatment of Data

Prior to beginning data analysis, any data set with missing points for the primary predictors or incomplete surveys was removed (*n* = 37). Three further data sets were removed due to incorrect engagement with the survey and duplications, thus the final sample for analysis was 298 participants.

### 3.2. Effect on Food Choice

**Figure 1:**
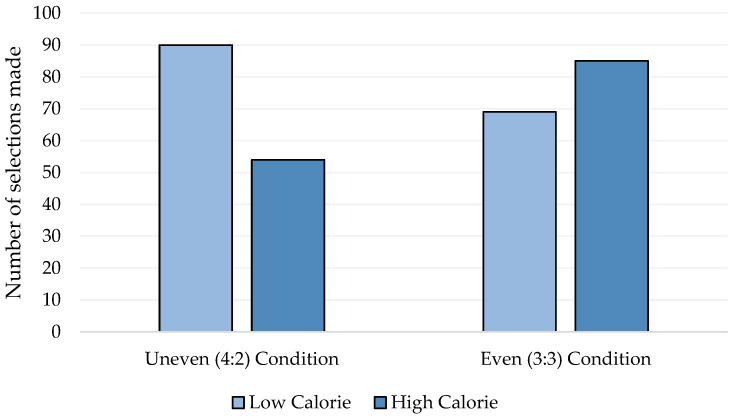
The frequency of low calorie (≤101 kcal) and high calorie (>201 kcal) selections made across the even (3:3 availability) and uneven (4:2 availability) conditions.

The binomial logical regression analysis revealed a nonsignificant predictive value of state and trait worry and rumination (all *p* > 0.10) on snack choice. However, the analysis revealed that there was significant predictive value of the experimental condition (*p* = 0.003). It was revealed that participants in the uneven condition were over twice as likely to opt for one of the lower calorie snacks over the high calorie snack in contrast to those in the even condition (OR: 2.03; CI: 1.27–3.25) (Figure 1). Finally, self-reported hunger was not found to be a significant predictor of snack choice (*p* = 0.137).

### 3.3. Effect on Calorie Consumption

The multiple regression model significantly predicted total hypothetical calorie intake from the food task paradigm, F(6, 291) = 4.25, *p* < 0.001 (adj. R^2^ = 0.062). State levels of hunger, rumination and worry were all found to be nonsignificant predictors to the model. However, trait levels of rumination (*p* < 0.001) and worry (p < 0.001), along with condition (*p* = 0.025), were all found to significantly contribute to the model. The regression coefficients for the predictors can be found in Table 2.

As can be seen from the coefficients in the table above, total calorie consumption was predicted to be 97.59 kcal higher in the even (3:3 availability) group relative to the uneven group (4:2 availability group). Regarding trait rumination, the slope coefficient suggests that for every single increase in the score on the brief RSS this was associated with an increased hypothetical consumption of 32.64 kcal. Conversely, each singular increase on the UB-PSWQ was associated with a hypothetical reduction by 33.99 kcal. All the other predictors included did not significantly contribute to the model (*p* > 0.10).

## 4. Discussion

The current study aimed to build upon a previous pilot investigation [30] in which we aimed to test whether the two facets of perseverative cognition, worry and rumination, would lead to poorer food choice as well as increased (hypothetical) calorie consumption; within two conditions that varied in availability of higher calorie snacks. In line with Hypothesis 1b, the results showed that an increased availability of low-calorie snacks made it twice as likely for an individual to opt for a lower calorie snack relative to the even availability condition. However, contrary to Hypothesis 1a, both state and trait rumination and worry showed not to significantly influence snack choice. Regarding hypothetical calorie consumption, both higher levels of trait rumination and an even availability of high and low-calorie snacks significantly predicted higher total calorie consumption on the food paradigm. Although, in conflict with initials predictions, higher levels of trait worry were found to be associated with a relative reduction in overall calorie consumption. These results led to the acceptance of Hypothesis 2b and the partial acceptance of Hypothesis 2a.

The observation that higher levels of trait rumination were a predictor of higher overall calorie consumption is in line with previous investigations [27,41]. Despite being unable to influence food choice, the increase in total calories regardless of the choice architecture employed may also tentatively suggest that behavioural interventions may be unable to mitigate the effects of rumination on the volume of unhealthy eating. This is likely to be a compensatory behaviour whereby increased consumption of sugary snacks is performed to provide a temporary anxiolytic effect [22,42]. This could then result in a negative feedback loop, as high sugar and fat-containing food can lead to higher levels of anxious and depressive symptoms in the long term [15,43], and, subsequently, further detrimental dietary behaviours [44]. This has led some researchers to propose that interventions to improve health and weight outcomes should actually be targeted towards tackling stress and emotional regulation [45,46], rather than employing common strategies such as calorie restriction alone. Although, in light of the nonsignificant influence of state rumination, this may tentatively suggest instead that higher levels of trait rumination leave an individual more susceptible to, or rather exacerbate, increased calorie consumption following a stressful or relevant environmental trigger. Whether momentary levels of rumination can instigate unhealthy eating behaviour in its own right remains to be elucidated. That said, Clancy et al. [29] found that brooding was still predictive of unhealthy snacking even when stress was included within the model. Further research should seek to expand this work into more ecologically valid environments whilst utilizing ecological momentary assessment (EMA) measurements. Additionally and/or alternatively, work could seek to explore the effects of rumination when induced experimentally. This would allow for a causal effect of rumination to be investigated further while allowing for exploration as to whether rumination increases calorie consumption in its own right or elevate consumption upon an appropriate environmental trigger.

The inverse association of higher worry and lower calorie consumption is at odds with the initial predictions and previous literature [47]. Why worry may lead to lower overall calorie consumption is unclear. It is worth noting, however, that eating responses to stress are not always uniform. For instance, some individuals have been found to eat less when stressed [22]. Moreover, given the focus on health and snack consumption within the current study, it is entirely possible that participants could have responded to enquiries regarding worry to be related to that of health. Indeed, worrying about one’s health is hypothesized to predict health-promoting behaviours and is a frequent inclusion in several social cognitive models within health psychology [48]. The more general repetitive negative thought process of worrying about future events has not been clearly associated with either health promoting or unhealthy eating behaviours to date [27]. This, of course, could be due to the small amount of literature available on this aspect of PC, and therefore, this result should still prompt further investigation to worry and eating behaviours.

Moving onto the impact of the choice architecture intervention, the results demonstrated that those in the condition with a higher availability of lower calorie snacks were twice as likely to opt for such in contrast to those with an even availability of low and high calorie snacks. What is more, this effect was not only found to improve food choice but also the of total number of calories consumed. This may suggest that such a behavioural devices might be effective in reducing impulsive food purchases of higher calorie snacks in favor of lower calorie choices. Although, to extend this conclusion to total calorie consumption should only be done so with caution. The regression model revealed that the total calorie difference between the two conditions was ~98 kcal, which is also approximately the difference between each of the low and high calorie snacks. This is of particular importance, given the observation that there were also no significant differences in the number of items chosen between the groups (see Table 1). In addition, in light of the significant influence of both worry and rumination within the final regression model, this may also suggest that reducing the availability of higher calorie snacks may be a limited intervention when combating negative emotion-related eating behaviours. However, the very interest in such an intervention is to acutely limit the consumption or purchase of adverse snack foods, which are typically obtained in an impetuous fashion in times of negative affect. It is worth exploring whether rumination, in particular, limits other behavioural “nudge” tactics to better understand the efficacy of these interventions to assist in achieving public health goals.

This study is not without its limitations. There are several factors that also influence food choice that were not measured here, namely whether the individual is currently undergoing some sort of dietary restraint. Indeed, individuals seeking to restrict their dietary choices (in terms of either food type and or macronutrient content) are well cited to be more likely to engage in poorer dietary choices in times of regular or sustained stress [19,49]. It could also be argued that the online nature of the study (in which no food was physically consumed by the participants) perhaps limits the conclusions that can be drawn. However, it is noteworthy that as food purchases become ever more accessible online (and or via mobile applications), this does still provide a valid indicator of the desire to order and consume food [30]. Other important considerations of the current food choice paradigm include those of the demand characteristics of the participants. In fact, the contrasting differences observed here from worry and rumination on calorie consumption may be influenced by the expectations of the participants. This affirms the need for further study within natural dining settings, where interventions and their aims are less discernible to the participants. Although, it is interesting to consider that positive approval ratings have been found to be as high as 90% for certain nudge tactics in real world settings [50]. This could suggest that even if individuals are aware of the nudges being employed, this will not decrease their efficacy. Whether this would apply to those experiencing high levels of rumination and or worry remains to be tested.

## 5. Conclusions

In summary, the current study has demonstrated that the two components of PC might function selectively to modulate eating behaviours. Moreover, despite observing that increasing variability of lower calorie snacks improves both food choice and total food calories consumed, this was found to be independent of the significant influence of the PC variables on the total number of hypothetical calories consumed. This may suggest that, despite its ability to acutely improve snack consumption, increasing the availability of lower calorie snacks may not be the most efficacious of interventions to reduce any PC-induced modulation of snack consumption. That said, the findings here are unable to shed light directly on whether trait rumination simply leaves an individual more susceptible to stress-induced changes to food consumption, or whether it is a direct catalyst of such behaviours. Future research should aim to further these findings in more ecologically valid settings and in individuals who are undergoing some sort of dietary restriction to better unpick the influence of both rumination and worry on snacking behaviours within nudge tactic paradigms.

## Figures and Tables

**Table 1:** Demographic information of the overall sample and the two study conditions. Due to data catchment errors, only complete datasets for each descriptive variable are reported here. The corresponding *n* is reported for each demographic factor for clarity.

		Even Condition (*n* = 154)	Uneven Condition (*n* = 144)	Overall (*n* = 298)
Age	*n*	154	144	298
	Mean	27.76	27.77	27.77
	SE mean	0.72	0.69	0.50
Gender	Female	106 (68.8%)	100 (69.4%)	206 (69.1%)
	Male	46 (29.9%)	41 (28.5%)	87 (29.1%)
	Other	2 (1.3%)	3 (2.1%)	5 (1.7%)
BMI	*n*	144	138	289
	Mean	24.41	23.50	23.96
	SE mean	0.44	0.41	0.4
Hunger	*n*	154	144	298
	Mean	42.43	38.22	40.33
	SE mean	2.04	2.10	2.07
	*n*	154	144	298
Number of Snacks	Mean	2.33	2.39	2.36
Selected	SE mean	0.13	0.13	0.13
State Rumination	*n*	154	144	298
	Mean	45.27	47.29	46.28
	SE mean	2.06	2.28	2.17
	*n*	154	144	298
State Worry	Mean	45.27	47.29	46.28
	SE mean	2.07	2.50	2.29
	*n*	154	144	298
Trait Rumination	Mean	11.75	11.54	11.65
	SE mean	0.29	0.31	0.30
Trait Worry	*n*	154	144	298
	Mean	9.59	9.32	9.46
	SE mean	0.27	0.28	0.28
Health Status	Poor	1 (.6%)	3 (2.19%)	4 (1.3%)
	Fair	14 (9.1%)	17 (11.8%)	31 (10.4%)
	Good	56 (36.4%)	47 (32.6%)	103 (34.6%)
	Very Good	63 (40.9%)	58 (40.3%)	121 (40.6%)
	Excellent	20 (13.0%)	19 (13.2%)	39 (13.1%)
Household Income	£0–£14,000	48 (31.2%)	50 (34.7%)	98 (32.9%)
	£14,001–£24,000	27 (17.5%)	27 (18.8%)	54 (18.1%)
	£24,001–£30, 000	19 (12.3%)	13 (14.0%)	32 (10.7%)
	£30,001–£40,000	16 (10.4%)	14 (12.1%)	30 (10.1%)
	£40,001–£80,000	25 (16.2%)	25 (20.6%)	50 (16.8%)
	£80,001+	19 (12.3%)	15 (10.4%)	34 (11.4%)

**Table 2:** Summary of results from the predictors for the multiple regression analysis for total calorie consumption from across the food task. * *p* < 0.05, *** *p* < 0.001.

Calorie Consumption	B	95% CI for B		SE	β	Sig
		Upper	Lower			
Constant	171.173	−33.569	375.916	104.028		0.101
Group	97.587	12.446	182.729	43.260	0.127	0.025 *
Hunger	0.456	−1.251	2.162	0.867	0.030	0.600
Trait Worry	−33.991	−51.621	−16.361	8.957	−0.293	0.000 ***
Trait Rumination	32.639	15.800	49.477	8.556	0.308	0.000 ***
State Worry	−1.174	−3.349	1.001	1.105	−0.081	0.289
State Rumination	1.088	−0.985	3.162	1.053	0.079	0.302

## Data Availability

The data presented in this article is available upon reasonable request from the corresponding author.

## Supplementary Materials

The following supporting information can be downloaded at: https://www.mdpi.com/article/10.3390/bs12030067/s1, S1: Paradigm and Stimuli; S2: Visual Analogue Scales.
