# Supplementary material for: Rumination and Worry Selectively Modulate Total Calorie Consumption within an Online, Nudge Tactic Paradigm
Source: Behav Sci (Basel). 2022 Mar 2;12(3):67. doi: 10.3390/bs12030067 (PMC8945422; doi:10.3390/bs12030067)
Supplement: Supplementary file 1 [file behavsci-12-00067-s001.zip › behavsci-1583089-supplementary.pdf]

S1: Paradigm and Stimuli

Uneven (4:2) condition illustration

"Please select the option that you would most like to consume right now."

Doughnut (50g) – 227Kcal	Chocolate chip cookie (19g) – 97kcal
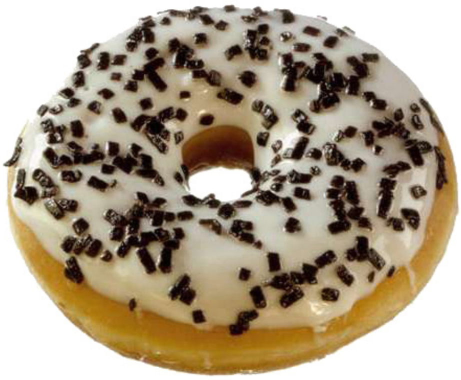	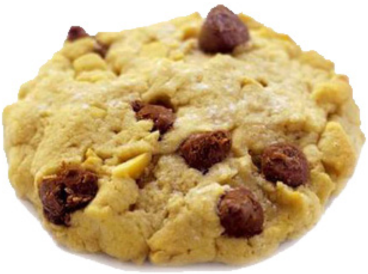
Marshmallows (30g) – 101kcal	Jelly Sweets (80g) – 274kcal
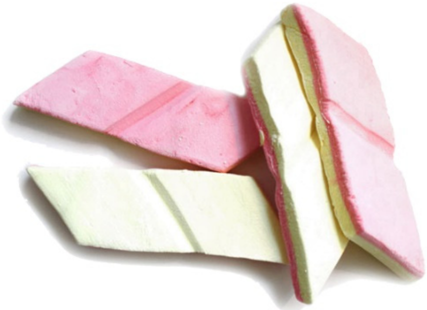	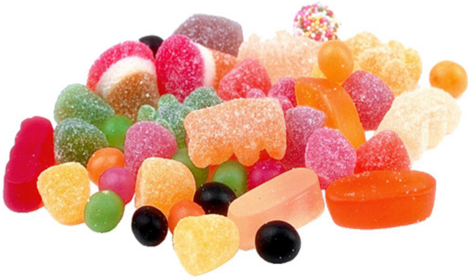
Liquorice Wheels (20g) – 75kcal	Marzipan Chocolate bite (19g) – 91kcal
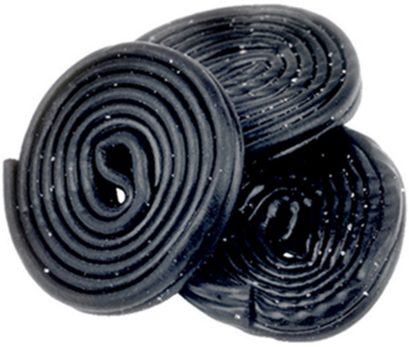	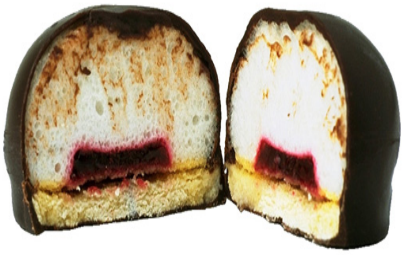

NB: Images were presented in a random order.

Below is the option that you chose in the previous selection. Now please indicate how many of this selection and serving that you would like to consume right now.

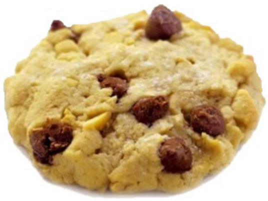

Chocolate Chip Cookie (19g) - 97Kcal

- ○ **1**
- ○ **2**
- ○ **3**
- ○ **4**
- ○ **5**
- ○ **6**
- ○ **7**
- ○ **8**
- ○ **More than 8. Please specify.**

Even (3:3) condition illustration

"Please select the option that you would most like to consume right now."

Jelly Sweets (80g) – 274kcal<br>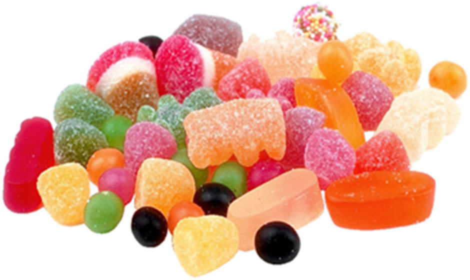	Marzipan Chocolate bite (19g) – 91kcal<br>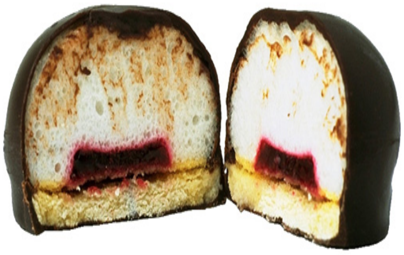
Marshmallows (30g) – 101kcal<br>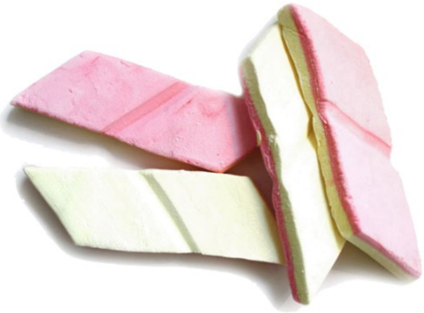	Doughnut (50g) – 227Kcal<br>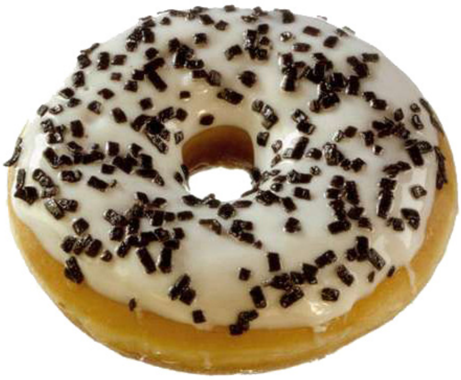
Chocolate chip cookie (19g) – 97kcal<br>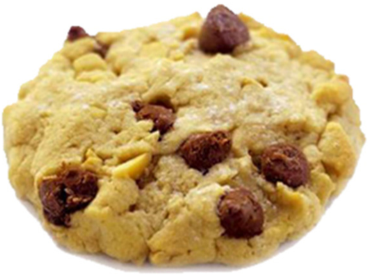	Cupcake (80g) – 275Kcal<br>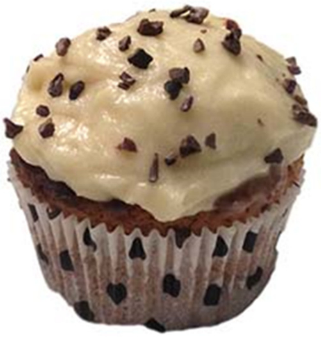

NB: Images were presented in a random order.

Below is the option that you chose in the previous selection. Now please indicate how many of this selection and serving that you would like to consume right now.

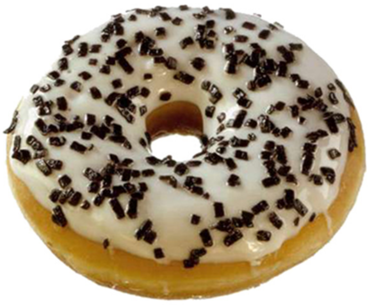

Doughnut (50g) – 227Kcal

- **1**
- **2**
- **3**
- **4**
- **5**
- **6**
- **7**
- **8**
- **More than 8. Please specify.**

Foodpic item numbers: Cookie = 0004, Doughnut = 0041, Marshmallows = 0147, Jelly Sweets = 0339, Liquorice Wheels = 0336, Marzipan Chocolate Bite = 0511 & Cupcake = 0672. Above images were used with permissions. For access and further details see here: https://sites.google.com/site/eatingandanxietylab/resources/food-pics

S2: Visual Analogue Scales

Hunger:

Please respond to the statement "How hungry are you?" on the slider. Consider how you are feeling at this very moment in time. Please note that the two ends of the scale represent the two extremes, with 0 denoting the least hunger that you have ever felt and 100 the most hungry that you have ever been in your entire life.

Not at all **Very much so**

0 **100**

State worry:

Today, how often have you tended to '*worry*' or focus on negative things that may occur or happen to you in the future? Please note that the two ends of the scale represent the two extremes, with 0 denoting the least that you have ever worried and 100 the most that you have ever worried in your entire life.

Not at all **Very much so**

0 **100**

State rumination:

Today, how often have you tended to '*ruminate*' or dwell over negative things that happened to you or upset you any time in the past? Please note that the two ends of the scale represent the two extremes, with 0 denoting the least that you have ever ruminated and 100 the most that you have ever ruminated in your entire life.

Not at all **Very much so**

0 **100**
